# Molecular Detection of *Acinetobacter* Species in Lice and Keds of Domestic Animals in Oromia Regional State, Ethiopia

**DOI:** 10.1371/journal.pone.0052377

**Published:** 2012-12-17

**Authors:** Bersissa Kumsa, Cristina Socolovschi, Philippe Parola, Jean-Marc Rolain, Didier Raoult

**Affiliations:** 1 Department of Parasitology, College of Veterinary Medicine and Agriculture, Addis Ababa University, Bishoftu, Ethiopia; 2 Aix Marseille Université, URMITE, UM63, CNRS 7278, IRD 198, Inserm 1095, Marseille, France; The University of Hong Kong, China

## Abstract

This study was conducted to determine the presence of *Acinetobacter* and *Rickettsia* species DNA in lice and *Melophagus ovinus* (sheep ked) of animals from Oromia Regional State in Ethiopia. From September through November 2011, a total of 207 cattle, 85 sheep, 47 dogs and 16 cats were examined for ectoparasites. Results of morphological identification revealed several species of ectoparasites: *Linognathus vituli (L. vituli)*, *Bovicola bovis (B. bovis)* and *Solenopotes capillatus (S. capillatus)* on cattle; *B. ovis* and *Melophagus ovinus (M. ovinus)* on sheep; and *Heterodoxus spiniger (H. spiniger)* on dogs. There was a significantly (p≤0.0001) higher prevalence of *L. vituli* observed in cattle than both *S. capillatus* and *B. bovis*. Molecular identification of lice using an 18S rRNA gene analysis confirms the identified lice species by morphological methods. We detected different *Acinetobacter* species among lice (11.1%) and keds (86.4%) including *A. soli* in *L. vituli* of cattle, *A. lowffii* in *M. ovinus* of sheep, *A. pittii* in *H. spiniger* of dogs, 1 new *Acinetobacter* spp. in *M. ovinus* and 2 new *Acinetobacter* spp. in *H. spiniger* of dogs using partial *rpoB* gene sequence analysis. There was a significantly higher prevalence of *Acinetobacter* spp. in keds than in lice (p≤0.00001). Higher percentage of *Acinetobacter* spp. DNA was detected in *H. spiniger* than in both *B. ovis* and *L. vituli* (p≤0.00001). Carbapenemase resistance encoding genes for blaOXA-23, blaOXA-24, blaOXA-58, blaNDM-1 and blaOXA-51 were not found in any lice and keds. These findings suggest that synanthropic animals and their ectoparasites might increase the risk of human exposure to zoonotic pathogens and could be a source for *Acinetobacter* spp. infections in humans. However, additional epidemiological data are required to determine whether ectoparasites of animals can act as environmental reservoirs and play a role in spreading these bacteria to both animal and human hosts.

## Introduction

Lice and sheep keds (*Melophagus ovinus*) are two of the most common and economically important ectoparasites of domestic animals worldwide. They are responsible for a wide range of health problems in domestic animals [1]. In infested animals they cause losses in productivity; due to anemia, loss of wool or hair due to scratching, biting and rubbing, disruption in feeding, hide and skin damage, secondary skin infections and damage, reduced newborn birth weights, abortion in pregnant animals, and damage to fences, equipment and buildings due to excessive rubbing and scratching [2], [3].

Several species of lice have been found on Ethiopian cattle; one species of chewing lice, *Bovicola* (*Damalinia*) *bovis*, and three species of sucking lice, including *Linognathus vituli* (the long-nosed cattle louse), *Solenopotes capillatus* (the little blue cattle louse) and *Haematopinus quadripertusus* (the cattle tail louse) [4]. On sheep, the biting lice *Bovicola (Damalinia) ovis*; one species of sucking lice, *Linognathus ovillus* and one species of the fly, *Melophagus ovinus*, are common ectoparasites [5], [6]. On goats, one species of biting lice, *Bovicola (Damalinia) caprae*, and the sucking lice *Linognathus stenopsis* have been reported [6], [7]. On dogs, the biting lice *Heterodoxus spiniger* and *Trichodectes canis* as well as one species of sucking lice, *Linognathus steosus*, were reported. In contrast, there are no reports available on the lice from cats in Ethiopia [8].

All the previous reports on the lice and sheep keds of animals in Ethiopia resulted from studies of other ectoparasites, specifically ticks and mange mites [6], [7], [9], [10], [11]. The only lice-specific study in Ethiopia was carried out by Kumsa and Bekele (2008) on the lice of cattle in the Endegagn district. Earlier studies focus on the epidemiology, species composition, distribution and impact of ectoparasites on the skin and hides of food animals. Studies on the role of lice and keds of domestic animals as vectors of pathogens of veterinary and medical importance have not yet been conducted in Ethiopia.

*Acinetobacter* species are Gram-negative coccobacilli bacteria commonly found in water, soil, mud, living organisms, vegetables, as well as in the feces, urine and skin of humans and animals [12], [13], [14], [15]. Currently, the genus comprises 23 validly named species and 12 genomic species. Owing to difficulty of precise identification of all members to the species level with advancement and development of new techniques in molecular methods, the taxonomy of the genus *Acinetobacter* has been continually revised [16], [17], [18]. Recently, formerly *Acinetobacter* genomic sp. 3 was renamed as *A. pittii, Acinetobacter* genomic sp. 13TU as *A. nosocomialis* and *Acinetobacter* genomic sp. G13 as *A.lwoffii* [16].

In humans, members of the genus *Acinetobacter* have emerged as opportunistic pathogens, and are frequently implicated in various types of infections, especially in immunocompromised individuals and intensive health care units [14] throughout the world. In tropical countries, they are associated with severe community-acquired infections [13]. They have the capability to survive for prolonged periods under a wide range of environmental conditions [13]. *A. baumannii* is described as the most common species in this genus frequently associated with outbreaks and has been repeatedly reported to develop a high level of resistance against all available classes of antimicrobial drugs in many parts of the world [12]. *A. pittii* is reported as the second most commonly isolated *Acinetobacter* species after *A. baumannii* in human patients [16], [17]. More recently other less known species such as *A. lwoffii* and *A. soli* have been associated with serious infections and considered as emerging pathogens [14], [19], [20]. For instance *A. lwoffii* has been associated with acute gastroenteritis in USA [21], multidrug resistant *A. lwoffii* in southern Thailand [22], *A. soli* outbreak as a cause of infection in neonatal intensive health care unit in Korea [23] and nosocomial bloodstream infections due to *A. pittii* in United States were reported.

In animals infection due to *Acinetobacter* species is considered as an emerging problem due to escalating in the number of reports from many countries of the world. Nosocomial infection by *A. baumannii* in dogs and cats in intensive care unit in Switzerland [24], infection due to *A. baumannii* in pets and horses in Switzerland [25], multidrug resistant *Acinetobacter* spp. in veterinary clinics in Germany [12], detection of *A. baumannii* in samples from cattle and pigs slaughtered for human consumption in major Scottish abattoirs [26], carbapenemase producing *Acinetobacter* spp. in cattle from France [27] and OXA-23 producing *Acinetobacter* spp. from faeces of horses in Belgium [28] are some of the current reports that notify the growing importance of *Acinetobacter* spp. in veterinary medicine mirroring the situation happening in human medicine.

In arthropods *Acinetobacter* spp. have been detected in different species including tsetse flies, sand flies, mosquitoes, fleas and ticks in many countries of the world. Details on *Acinetobacter* spp. detected in arthropods, species of vectors and methods of detection is presented (Table 1). Also, recently several investigators detected *A. baumannii* in both body and head lice of humans from some countries [15], [29], [30], [31]. Despite the worldwide distribution and great economic significance of these ectoparasites, information is not available on the occurrence of *Acinetobacter* species in the lice and flies found on domestic animals. In addition, there is little known about *Rickettsia* species in the lice and flies of domestic animals in Ethiopia. Therefore, the current study investigated the presence of these bacteria in arthropods of domestic animals in six districts in Oromia Regional State, Ethiopia.

**Table 1:** Summary of *Acinetobacter* spp. detected in various species of arthropods from different countries of the world.

*Acinetobacter s*p.	Arthropod sp.	Detection method	Country	Reference
*A. radioresistans*	*Ctneocephalides felis*	Culture and PCR	Australia	[53]
*A. johnsonii*	*Ct. felis*	Culture and PCR	Australia	[53]
*A. junii*	*Ct. felis*	Culture and PCR	Australia	[53]
*A. lwoffii*	*Ixodes holocyclus*	Culture and PCR	Australia	[53]
*A. lwoffii*	*Boophilus microplus*	Culture and PCR	Australia	[53]
*A. johnsonii*	*Boophilus microplus*	Culture and PCR	Australia	[53]
*A. junii*	*Boophilus microplus*	Culture and PCR	Australia	[53]
*A. radioresistans*	*Boophilus microplus*	Culture and PCR	Australia	[53]
*A. baumannii*	*Lutzomyia longipalpis*	Culture and PCR	Brazil	[39]
*A. juni*	*Culex quinquefasciatus*	PCR	India	[54]
*A. calcoaceticus*	*Culex quinquefasciatus*	PCR	India	[54]
*A. calcoaceticus*	*Anopheles stephensis*	PCR and biochemical	Iran	[55]
*A. calcoaceticus*	*Anopheles maculipennis*	PCR and biochemical	Iran	[55]
*Acinetobacter s*p.	*Lutzomyia longipalpis*	PCR	Brazil	[56]
*Acinetobacter s*p.	*Bemisia tabaci*	PCR	Iran	[44]
*Acinetobacter s*p.	*Glossina palpalis palpalis*	Culture and PCR	Cameroon	[57]
*Acinetobacter s*p.	*Bactericera cockerelli*	PCR	USA	[42]
*A. genomosp. 3*	*Aedes albopictus*	PCR	Madagascar	[58]
*A. genomosp. 13U*	*Aedes aegypti*	PCR	Madagascar	[58]
*A. baumannii*	*Glossina palpalis palpalis*	Culture and PCR	Angola	[40]

## Materials and Methods

### Study areas and animals

Lice and *Melophagus ovinus* were collected from indigenous cattle, sheep and dogs in six different districts in Oromia Regional State, Ethiopia: Asalla, Walmara, Shano, Ada'a, Bedele and Gachi. The districts are located in 5 zones in the central, southeast and southwestern parts of the country, with various climates and agroecology (Table 2). Ectoparasite collections were performed from September through November of 2011. A long rainy season from July to September, a short rainy season from March to May and a dry season from November to April prevail in all the study districts. In addition, October is a post rainy month with only few days of raining while November is a month during which the dry season commences hence is without raining or rarely for some days. The farming system in all the districts is characterized by a mixed crop-livestock production system. The livestock in the study areas are traditionally managed under extensive production systems [32].

**Table 2:** Description of study districts and number of study animals in Oromia Regional State.

District	Zone	Km from Addis Ababa	Agroecology	Altitude in m.a.s.l	Coordinates	Annual rainfall in mm	Av. Annual Temp in °C	No. Animals examined
Asalla	Arsi	175 southeast	Highland	2500	7°56′58′.57′′N 39°8′23.42′′ E	2000–4000	20–30	Sheep = 39
Shano	North Showa	70 Northeast	Highland	2861	9°20.00.00′′N 39°18′00.00′′E	945.4	6.1–18.5	Cattle = 38 Sheep = 19
Walmara	West Showa	45 west	Highland	2500	9°7′50.64′′N 38°28′38.46′′E	1060	4.6–23.3	Cattle = 42
Ada'a	East Showa	47 east	Midland	1911	8°44′37.69′′N 38°59′19.28′′E	1911	13–26.5	Cattle = 53 Dogs = 47 Cats = 16
Bedele	Illubabora	483 southwest	Midland	1974	8°,27′1.76′′N 36°21′5.08′′E	1400	12.5–27.5	Cattle = 32 Sheep = 21
Gachi	Illubabora	460 southwest	Midland	1751	9°14′2.57′′N 35° 4′48.46′′E	1300	13–28	Cattle = 42 Sheep = 6

m.a.s.l = meters above sea level; Av. = average; °C =  degrees Celsius.

### Collection and morphological identification of ectoparasites

**Figure 1:**
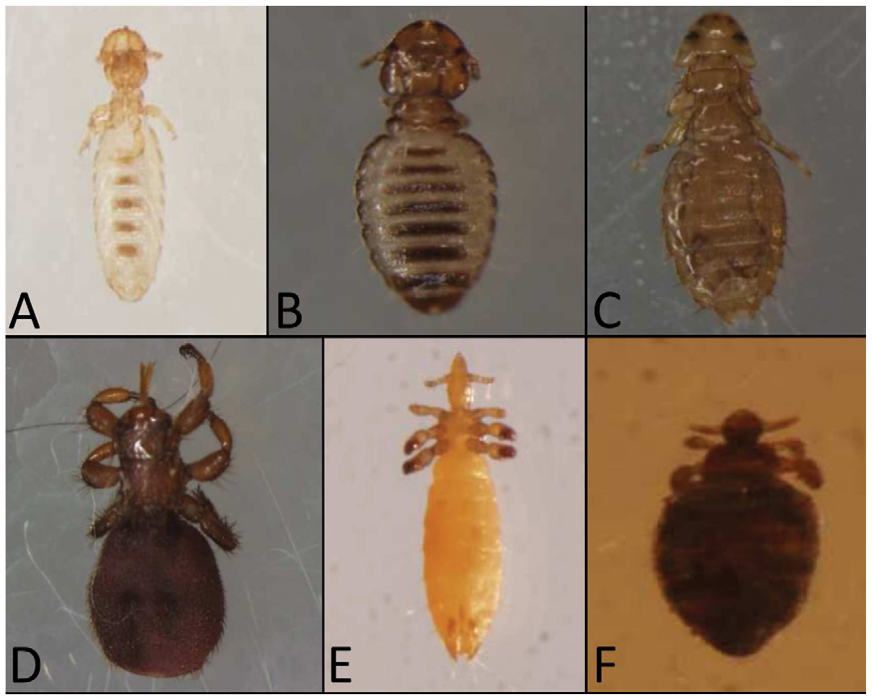
Photographs of morphologically identified 5 species of lice and *Melophagaus ovinus* collected from domestic animals. A, *Bovicola ovis* of sheep; B, *Bovicola bovis* of cattle; C, *Heterodoxus spiniger* of dog; D, *Linognathus vituli* of cattle; E, *Solenopotes capillatus* of cattle; F, Melophagus ovinus (sheep ked) of sheep.

Lice and flies were carefully removed manually, using forceps or by hand, to avoid any damage to the body and were then placed in vials containing 70% ethanol for subsequent identification. In the case of lice collected from dogs, the dog's body was brushed for ten minutes with a flea comb as previously described [8]. All lice and flies from the same animal were put in the same vial and transported to the Laboratory of the World Health Organization Collaborative Center for Rickettsial Diseases and Arthropod-borne Bacterial Diseases located in Marseille, France. Morphological identification of ectoparasites and molecular studies were performed from the end of 2011 through 2012. All of the ectoparasites were identified to the species level using a microscope and the morphological identification keys described [1], [3]. Photographs of the dorsal and ventral body parts of each ectoparasite were captured (Fig. 1), and the number and sex of each louse and fly was determined.

### Molecular identification of ectoparasites

Prior to DNA extraction, each specimen was rinsed twice in sterile water for 15 minutes and then dried on sterile filter paper. Each specimen was longitudinally cut into two equal halves. Genomic DNA was extracted from each specimen using the QIAamp DNA tissue extraction kit (Qiagen, Hilden, Germany) as per the instructions of the manufacturer. DNA from each ectoparasite was eluted in 200 µl of TE buffer and stored at −20°C under sterile conditions to preclude any contamination until the sample was used for PCR. The second half of each louse and fly was kept at −80°C as a backup sample.

For molecular species identification, DNA samples of 3 to 8 randomly selected individual lice per species were subjected to standard PCR in an automated DNA thermal cycler to amplify a fragment of the 18S rRNA gene as described [33]. The PCR was carried out in a Peltier PTC-200 model thermal cycler (MJ Research Inc, Watertown, Mass.). The amplified products were detected by electrophoresis on 2% agarose gels in TBE 0.5× buffer, stained with ethidium bromide and visualized using ultraviolet (UV) transillumination. A DNA molecular weight marker (Boehringer-Mannheim VI, Germany) was used to estimate the size of the products. The positive controls consisted of one sample from *Pediculus humanus capitis* collected in Mali and one sample of *P. humanus humanus* collected in Algeria. These samples were included in the PCR assay, and sterile water was used as negative control.

The PCR products were cleaned of excess primers and nucleotides using a QIAquick Spin PCR Purification Kit (Qiagen) as per instructions of the manufacturer. Purified DNA was sequenced using the BigDye Terminator Cycle Sequencing Ready Reaction Kit (ABI PRISM, PE Applied Biosystems, Foster City, CA). All obtained sequences were assembled and edited using Chromas Pro1.34 (Technelyium Pty. Ltd., Tewantin). The sequences of the 18S rRNA genes were then subjected to BLAST analysis to determine similarities to those available in GenBank and to construct a phylogenetic tree using Mega 5 software (Molecular Evolution Genetic Analysis; The Biodesign Institute, Tempe, AZ).

### Molecular detection of *Acinetobacter* and *Rickettsia* species

All the specimens were individually tested for the presence of *Acinetobacter* species DNA targeting the *rpoB* gene [30] by real-time quantitative (q) PCR as per the instructions of the manufacturer (Applied Biosystems, Foster City, CA). In addition, the ectoparasite DNA was tested for spotted fever group *Rickettsia* with primers targeting the *glt*A gene specific for this group [34] and for typhus group *Rickettsia* with primers targeting the Rpr274P gene, which encodes a hypothetical protein [35]. Sterile water was used as negative control; *A. baumannii*, *R. montanensis* and *R. typhi* DNA were used as positive controls. The samples were considered positive when cycle thresholds (Ct) were <35.

### Molecular detection of carbapenemase encoding genes

All the DNA of lice (n = 82) and flies (n = 19) positive for *Acinetobacter* species were tested for the presence of carbapenemase encoding genes by qPCR targeting blaOXA-23, blaOXA-24, blaOXA-58 and blaNDM-1 using primers, probes and all conditions as has been described before [15], [36]. In addition, a total of 32 lice and 10 flies with sufficient amount of DNA were evaluated for carbapenemase encoding genes by standard PCR targeting blaOXA-51 and blaOXA-23 with primers and all conditions as described before [15].

### Molecular identification of *Acinetobacter* spp. by partial *rpoB* gene

A total of 32 lice and 10 keds DNA of sufficient amount and positive for *Acinetobacter* spp. by qPCR were further subjected to standard PCR targeting partial *rpoB* gene (zone 1) to identify *Acinetobacter* spp. using the primers and all conditions as described before [17]. Sterile water and *A. genomosp* DNA were used as negative and positive controls, respectively. Detection of amplified products, cleaning of excess primers and nucleotides from DNA, sequencing, assembling and edition of sequences, BLAST analysis and *rpoB* gene phylogenetic tree construction with Maximum likelihood statistics of Mega 5 were all performed using similar methods as described for 18S rRNA gene for lice above.

### Ethical statement

Ethical approval for the collection of lice and flies from domestic animals was obtained from the animal research ethics board (Agreement # 14/160/550/2011) of the College of Veterinary Medicine and Agriculture of Addis Ababa University. All necessary oral permits were obtained for the described field studies, including permission of administration and agricultural office of each Ethiopian district and from each animal owner. Ectoparasite collections are not harmful and are not against the welfare of animals. No collection had been done from privately-owned, wildlife, national park or other protected areas and endangered or protected species.

### Data analysis

Microsoft Excel was used for data management. Descriptive statistics such as percentages and means were employed to summarize the proportions of infestations with lice and keds. Statistical analysis was performed with EpiInfo^TM^7 and a P-value of <0.05 was considered significant.

## Results

### Morphological identification of ectoparasites

A total of 207 cattle, 85 sheep, 47 dogs and 16 cats in six districts were examined for the presence of lice and sheep keds (Table 2). The results of the morphological identification of the collected lice and flies from infested animals revealed a total of 408 *Linognathus vituli*, 4 *Bovicola bovis* and 3 *Solenopotes capillatus* from cattle; 22 *Heterodoxus spiniger* from dogs; 298 *Bovicola ovis* and 22 *Melophagus ovinus* from sheep (Fig. 1). Furthermore, the study showed that out of all of the examined cattle, 19.3% (40/207) were infested with *L. vituli*, 0.5% (1/207) with *S. capillatus* and 0.5% (1/207) with *B. bovis*. In cattle, there was a significantly higher prevalence of *L. vituli* than both *S. capillatus* and *B. bovis* (p≤0.0001). Of the total examined sheep, 48.2% (41/85) were infested with *B. ovis* and 21.05% (4/19) were positive for *M. ovinus*. In addition, 19.1% (9/47) of the examined dogs were infested with *H. spiniger*. Alternatively, lice were not detected on any of the cats studied.

### Molecular identification of ectoparasites

**Figure 2:**
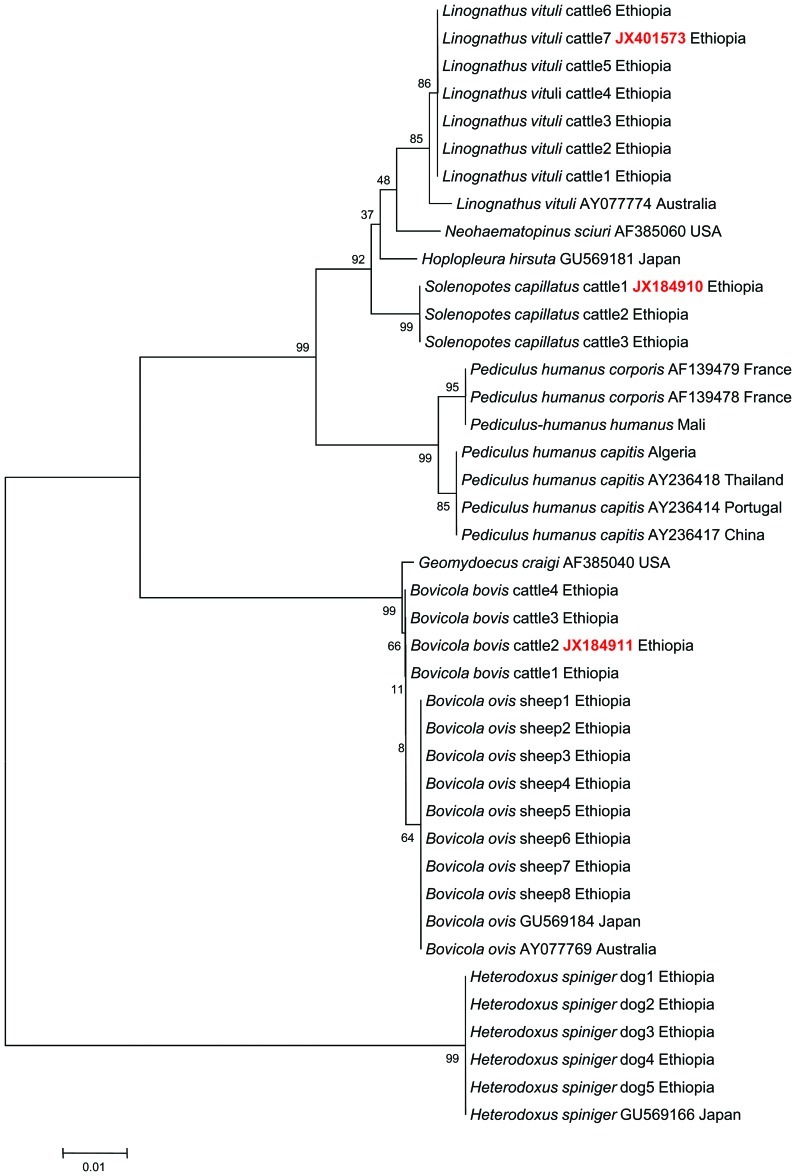
Phylogenetic tree based on 18S gene sequences of lice species collected from domestic animals. Accession numbers in red color are 18S gene sequence of lice of animals from Ethiopia recently deposited in the GenBank. Minimum evolution method was used to build the phylogentic tree. Bootstrap values are indicated at the nodes.

Molecular identification of the lice based on the 18S rRNA gene analysis was used to confirm the species of lice identified by morphological methods. A BLAST analysis of 18S rRNA gene sequences of lice from dogs morphologically identified as *H. spiniger* (n = 5) showed 100% (509/509) similarity to the GenBank reference of *H. spiniger* collected from Japan (GU569166) (Fig. 2). Likewise, a BLAST analysis of 18S rRNA gene sequences of lice that were collected from cattle and were morphologically identified as *L. vituli* (n = 7) showed 99.3% (553/557) similarity to *Linognathus vituli* collected in Australia (GenBank Access. No. AY077774). Four mutations were detected between our sequence and the reference (AY077774) at 283 bp (T-G), 327 bp (T-A), 352 bp (T-C), and 420 bp (T-C). This sequence was submitted to GenBank under accession number JX401573.

Interestingly, a sequence analysis of the 18S rRNA gene of 3 lice from cattle morphologically identified as *Solenopotes capillatus* showed 92.2% (536/581) homology with the GenBank sequence of *Neohaematopinus sciuri* (AF423798) and 92% (520/565) homology with *Hoplopleura hirsute* collected in Japan from the cotton rat *Sigmodon hispidus* (GU569181).

A BLAST analysis of the 18S rRNA gene of 4 *Bovicola bovis* lice from cattle showed 99.8% (494/495) similarity to *B. ovis* (GenBank Access No. GU569184) and 99.4% (510/513) similarity to *Geomydoecus craigi* (GenBank Access No. AF385040) (Fig. 2). There are no 18S rRNA gene sequences available in GenBank for *S. capillatus* and *Bovicola bovis*. Thus, these two sequences were submitted to GenBank under accession numbers JX184910 and JX184911, respectively. An analysis of the 18S rRNA gene of *Bovicola ovis* (n = 8) from sheep revealed 100% (495/495) similarity with *Bovicola ovis* detected in Japan and Australia with the sequences GU569184 and AY077769, respectively.

An analysis of the 18S rRNA gene from *Pediculus humanus humanus* (n = 1) from Mali revealed 99.4% (496/499) similarity with *Pediculus humanus corporis* detected in France with Accessions Nos. AF139479 and AF139478, respectively. An analysis of the 18S rRNA gene from *Pediculus humanus capitis* (n = 1) from Algeria revealed 99.8% (518/520) similarity with the *Pediculus humanus capitis* detected in Thailand, Portugal and China with Accessions Nos. AY236418, AY236414 and AY236417, respectively.

A phylogenetic tree was constructed from the 18S rRNA gene sequences of lice collected in this study and the most similar sequences in the GenBank using the Neighbor-joining statistics of Mega 5. *H. spiniger* reference lice and our lice sample from dogs that belong to the family Boophidae in the suborder Amblycera formed one group on the phylogenetic tree (Fig. 2). Reference *Linognathus vituli* and our lice sample from cattle that belong to the family Linognathidae under the suborder Anoplura clustered together. The 3 individual *S. capillatus* lice from cattle, which shared the same family and suborder with *L. vituli*, clustered near the cattle louse. Both the control and the reference *P. humanus capitis* and *P. humanus humanus* isolated from human head and body lice, belonging to the family Pediculidae under the suborder Anoplura, formed a separate group on the phylogenetic tree that was close to *L. vituli* from cattle. The *Bovicola ovis* reference lice and our lice sample from sheep that belong to the family Trichodectidae in the suborder Ischnocera clustered in a separate group close to the four *B. bovis* cattle lice, which belong to the same genus, family and suborder (Fig. 2).

### Detection of *Acinetobacter* and *Rickettsia* species in lice and *M. ovinus*

We detected *Acinetobacter* spp. in a total of 82 lice (11.1%) and 19 flies (86.4%) (Table 3). In our study, there was a significantly higher prevalence of *Acinetobacter* spp. in flies than in lice (82/735 vs. 19/22; p≤0.00001). The study showed that a higher percentage of *Acinetobacter* spp. DNA was detected in *H. spiniger* of dogs than in *B. ovis* and *L. vituli* (15/22 vs. 19/298; 47/408; p≤0.00001). The prevalence of *Acinetobacter* spp. in *L. vituli* collected from cattle was significantly higher than in *B. ovis* from sheep (47/408 vs 19/298; p = 0.02). *Acinetobacter* spp. was not detected in any of the 4 *Bovicola bovis* collected from cattle (Table 3).

**Table 3:** Percentage of *Acinetobacter* spp. detected by qPCR in lice and flies collected from domestic animals in six districts in Oromia.

District (Number of positive samples for *Acinetobacter* spp./Number of tested samples) (%)							
Lice spp.	Asalla (%)	Shano (%)	Walmara (%)	Ada'a (%)	Bedele (%)	Gachi (%)	Total (%)
*B. ovis*	13/234(5.5)	5/57(8.8)	-	-	1/7(14.3)	-	19/298(6.4)
*L. vituli*	-	7/51(13.7)	10/93(10.7)	4/84(4.8)	1/12(8.3)	25/168(14.9)	47/408(11.5)
*S. capillatus*	-	-	-	1/3(33.3)	-	-	1/3(33.3)
*B. bovis*	-	0/4(0)	-	0/0 (0)	-	-	0/4(0)
*H. spiniger*	-	-	-	15/22(68.2)	-	-	15/22(68.2)
**Total lice**							**82/735(11.1)**
Fly species							
*M. ovinus*	-	19/22(86.4)	-	-	-	-	19/22(86.4)
**Total flies**							**19/22(86.4)**

Results of our study revealed that out of the total lice infested animals, 88.9% (8/9) of dogs were infested with *H. spiniger*, 45% (18/40) of cattle were infested with *L. vituli* and 34.4% (14/41) of sheep were infested with *B. ovis* and harbored at least 1 louse positive for *Acinetobacter* spp. (Table 4). Similarly, *Acinetobacter* spp. was detected at least in one *M. ovinus* in all the 4/4 (100%) infested sheep. Highest proportion of *Acinetobacter* spp. was observed in cattle infested with *L. vituli* in Walmara and Gachi districts whereas highest percentage of *Acinetobacter* spp. was noted in sheep infested with *B. ovis* in Shano and Asalla districts (Table 4).

**Table 4:** Proportion of infested animals positive for *Acinetobacter* spp. in lice and keds by qPCR in six districts in Oromia.

District	No. animals positive for *Acinetobacter* spp./No. animals infested with lice or fly					
	*B. ovis* (Sheep)	*L. vituli* (Cattle)	*S. capillatus* (Cattle)	*B. ovis* (Cattle)	*H. spiniger* (Dog)	*M. vinus* (Sheep)
Asalla	9/29 (31.0%)	-	-	-	-	**-**
Walmara	-	6/9 (66.7%)	-	-	-	-
Shano	4/10 (40%)	2/9 (22.2%)	-	0/1	-	4/4 (100%)
Ada'a	-	3/9 (33.3%)	1/1 (100%)	-	8/9 (88.9%)	-
Bedele	1/2(50%)	1/4 (25%)	-	-	-	-
Gachi	-	6/9 (66.7%)	-	-	-	-
**Total**	**14/41(34.4%)**	**18/40 (45%)**	**1/1 (100%)**	**0/1**	**8/9 (88.9%)**	**4/4 (100%)**

A molecular investigation of the 735 lice collected from domestic animals and 22 *Melophagus ovinus* collected from sheep using qPCR did not produced any positive results for either spotted fever or typhus group *Rickettsia* species.

### Molecular identification of *Acinetobacter* spp

**Figure 3:**
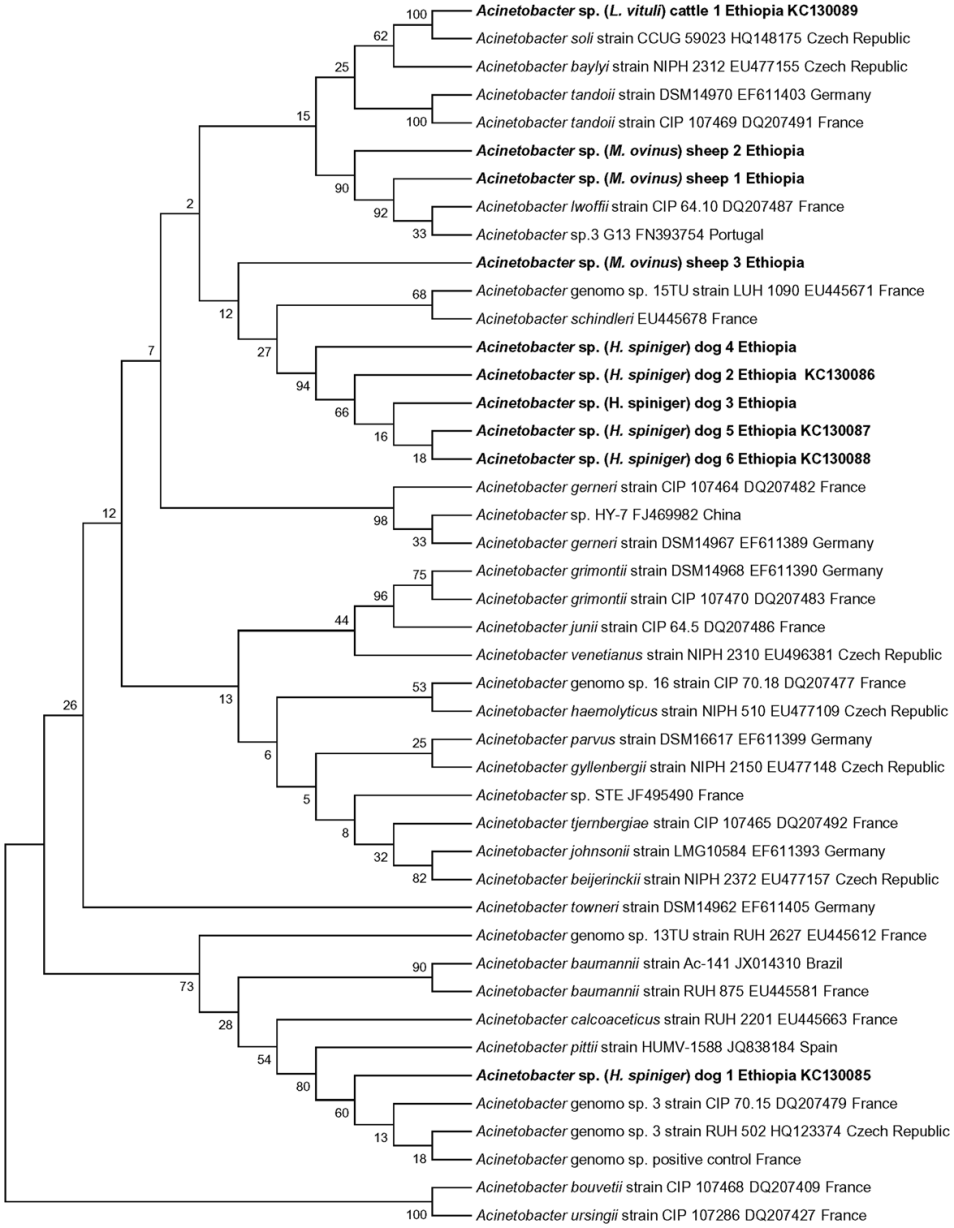
Phylogenetic tree based on partial *rpoB* gene sequences of *Acinetobacter* species. Maximum Likelihood method was used to build the phylogentic tree. Bootstrap values are indicated at the nodes. **Bold** indicates the taxonomic position of *Acinetobacter* species identified in this study.

We succeeded in the amplification of 350 bp fragment of partial *rpoB* gene from a total of 10 samples including one from *L. vituli* of cattle, 3 from *M. ovinus* of sheep and 6 from *H. spiniger* of dogs (Table 5). BLAST analysis of partial *rpoB* gene sequence and *rpoB* phylogenetic tree showed the presence of *A. soli* in *L. vituli* of cattle, *A. lwoffii* in *M. ovinus* of sheep, 1 new *Acinetobacter* sp. in *M. ovinus* of sheep, *A. pittii* in *H. spiniger* of dogs and 2 new *Acinetobacter* sp. in *H. spiniger* of dogs (Table 5 and Fig. 3). Five of these 10 sequences were submitted to GenBank under accession numbers KC130085-89, respectively.

**Table 5:** Summary of BLAST analysis of partial *rpoB* gene sequences obtained from lice and keds of domestic animals in six districts in Oromia, Ethiopia.

Lice/fly spp	Host spp	Length (bp)	Nearest match in GenBank	% similarity
*H. spiniger*	Dog	383	*Acinetobacter* genomosp. 3 (DQ207479)	99.2%
*H. spiniger*	Dog	351	*Acinetobacter* sp.Hy-7 (FJ469982)	90.8%
*H. spiniger*	Dog	391	*Acinetobacter* sp.Hy-7 (FJ469982)	90.7%
*H. spiniger*	Dog	310	*Acinetobacter* sp. Hy-7 (FJ469982)	86.5%
*H. spiniger*	Dog	387	*Acinetobacter* sp. Hy-7 (FJ469982)	90.5%
*H. spiniger*	Dog	388	*Acinetobacter* sp. Hy-7 (FJ469982)	90.5%
*L. vituli*	Cattle	381	*Acinetobacter soli* (HQ148175)	98.9%
*M. ovinus*	Sheep	365	*Acinetobacter* sp.G13 (FN393754)	98.9%
*M. ovinus*	Sheep	293	*Acinetobacter* sp.G13 (FN393754)	94.39%
*M. ovinus*	Sheep	328	*Acinetobacter* sp-Hy-7 (FJ469982)	90.2%
*P. h. humanus* (Control)	Man	383	*Acinetobacter* genomosp.3 (DQ207479)	99.2%

### Detection of carbapenemase encoding genes in *Acinetobacter* species

A molecular investigation of 82 lice and 19 keds positive for *Acinetobacter* spp. by qPCR did not produced any positive results for blaOXA-23, blaOXA-24, blaOXA-58 and blaNDM-1 genes encoding for carbapenemase resistance. Likewise, investigation of 32 lice and 10 keds DNA by standard PCR never produced any positive results for both blaOXA-23 and blaOXA-51 genes encoding for carbapenemase resistance.

## Discussion

In this study, we detected, for the first time, *Acinetobacter* spp. in the lice and flies of animals from Ethiopia. In addition, we obtained two new sequences of 18S rRNA genes from *Bovicola bovis* and *Solenopotes capillatus* collected from cattle. We believe that the results of this study are valid, as all of the negative control samples produced negative results and all the positive control samples tested positive for both *Acinetobacter* spp. and *Rickettsia* spp. detection in the lice and flies of these animals. These findings confirmed that our molecular study conditions precluded any accidental cross-contamination of samples during the study period. Similarly, in our 18S rRNA gene study of lice, we obtained sequences from both positive controls, *Pediculus humanus capitis* and *Pediculus humanus humanus*, confirming the appropriateness of our working conditions.

In the study, the species of lice morphologically identified as *L. vituli*, *B. bovis* and *S. capillatus* from cattle; *H. spiniger* from dogs; and *B.ovis* from sheep (Fig. 1) were confirmed molecularly using 18S rRNA gene sequences. The 18S rRNA gene has been used previously as an important tool to investigate human lice phylogeny [33], to study the evolution of sucking lice [37] and to study the phylogeny of lice [38]. The phylogenetic tree constructed from the 18S rRNA gene sequences of the collected lice and reference lice in the GenBank presented here supports the information from previous studies [37], [38].

In this study, we sequenced the 18S rRNA genes of *B. bovis* and *S. capillatus* from cattle for the first time. *B. bovis* is the only biting lice species in cattle, and it has important morphological features such as a rounded head, reddish-brown color and dark transverse bands on the abdomen [1] (Fig. 1). *S. capillatus* from cattle are the smallest sucking lice, with morphological characteristics that include a hexagonal sterna plate on the thorax, a weak front pair of legs and prominent abdominal tubercles [3]. In Ethiopia, both species are typically reported with low prevalence [4]. The finding in this study that there was a significantly (p≤0.0001) higher prevalence of *L. vituli* than both *S. capillatus* and *B. bovis* in cattle is in agreement with previous work [4]. Our findings in sheep, dogs and cats of this study are also in line with previous reports [5], [6], [8].

The observation that 11.1% of the lice in the current study were found to contain *Acinetobacter* spp. is lower than the earlier report of 47% in human head lice and 71% human body lice from Ethiopia [31], 21% in human body lice [29] and 33% in human head lice from Paris [30]. Recently, a low prevalence (4%) of *A. baumannii* in head lice was reported from Senegal [15]. However, the overall percentage of 86.4% (19/22) of *Acinetobacter* spp. detected in *M. ovinus* of sheep in our study is greater than that found in the aforementioned reports. The variation among study districts in the percentage of *Acinetobacter* spp. in lice and keds in infested animals is most probably attributed to differences in agroecology, animal management and factors like age, sex, physiological status or presence of other concurrent diseases that may favor infection of lice or the animal by the bacteria. This observation coincides with the findings of differences in the prevalence of infection in lice of humans by *A. baumannii* among different study areas with different altitudes in south western Ethiopia [31].

In line with our findings, *Acinetobacter* spp. were detected in many species of arthropods in different parts of the world (Table 1). For instance, *Acinetobacter* spp. have been detected in 13% of *Lutzomyia longipalpis* (sand flies) in Brazil [39], 7.7% of *Glossina palpalis palpalis* (tsetse fly) in Angola [40], 8.7% in the gut of the *Prionoplus reticularis* larvae (wood feeding beetle) in New Zealand [41], up to 75% in *Bacterria cockerelli* (potato psyllid) in the USA [42], 18.9% in chewing lice of pocket gophers in the USA [43] and 1% in *Bemisia tabaci* (tobacco whitefly) in India [44]. Moreover, *A. baumannii* have been detected with a prevalence of 5.1% in the feces of domestic animals in Senegal [15].

Most earlier investigators suggested that it is still yet not exactly determined how both body and head human lice acquired *A. baumannii* infection [30], [31]. However, some authors argued undiagnosed transient *A. baumannii* bacteremia in infested patients as a source of infection for body lice but they stated it is not possible to rule out the possibility of acquiring *A. baumannii* infection in human body lice from external environmental contamination [29]. Other investigators pointed out that bacterial spp. such as *Acinetobacter* abundant in the environment reside in the gut of several species of arthropods as transient or natural flora [39] acquired commonly by vertical transmission [40] and also are maintained and spread by several mechanisms of horizontal transmission including mating, cofeeding, or contact with contaminated faeces [41], [42], [44]. Having all these facts in mind and due to the ubiquitous nature of *Acinetobacter* spp. in the environment including on the skin and in the faces of animals [15] lice and keds possibly had acquired *Acinetobacter* spp. infection from the skin, faces or transient *Acinetobacter* spp. bacteremia of their host animals. Moreover, it is not possible to rule out infection of lice and keds by vertical route and also probably animals may play a role as reservoirs for these bacteria.

We did not detect *Rickettsia* DNA in the lice and flies in our study. This finding contrasts with previous work that reported detecting *R. helvetica* in *M. ovinus* from sheep, in *Linognathus stenopsis* from goats, and *Rickettsia* spp. in *Haematopinus eurysternus* from cattle in Hungary [45], [46].

The absence of any positive results for blaOXA-23, blaOXA-24, blaOXA-58, blaNDM-1 and blaOXA-51 genes encoding for carbapenemase resistance by both qPCR and standard PCR in *Acinetobacter* spp. in the lice and keds of domestic animals in our study is in line with the previous finding of absence of resistance to several antimicrobials in *A. soli* in intensive health care units of neonates in Brazil [23], full susceptibility to several antibiotics of *A. lwoffii* from acute gastroenteritis in USA [21] and absence of blaOXA-like genes encoding for carbapenemase resistance in *A. baumannii* from faeces of domestic animals in Senegal [15]. On the other hand our findings contrast the previous multidrug resistance in *A. baumannii* reported from several countries of the world [12], [36], [47]. This finding also contradicts the observations of multidrug-resistant isolates of *Acinetobacter* spp. from a range of environmental sources in South Korea [20] and the presence of resistance to antibiotics in *A. baumannii* and other *Acinetobacter* spp. from blood cultures in Norway [48]. This variation is most probably attributed to differences in strains of the bacteria among various studies and other many factors contributing for emergence of resistance.

*Acinetobacter* spp. identification study from DNA of lice and keds of animals in Oromia uncovered the occurrence of 3 previously described species and 3 new *Acinetobacter* spp. (Table 5 and Fig. 3). All the 3 previously described species, we detected: *A. soli* from *L. vituli* of cattle, *A. lowffii* from keds of sheep and *A. pittii* from *H. spiniger* of dogs with high nucleotide sequence identities of 98–100% with their respective reference species in the GenBank (Table 5). This finding supports the criteria established for *Acinetobacter* spp. identification using partial *rpoB* gene sequence analysis [17], [18]. In line with our observation higher predominance of other *Acinetoabacter* spp. (24.8%) than *A. baumannii* (only 8.8%) from human blood culture isolates was recently reported from Norway [48]. Furthermore, these species are nowadays reported to cause various types of human infections worldwide [21], [23], [49], [50], [51] and are implicated as emerging *Acinetobacter* spp.

We also identified two new *Acinetobacter* species from *H. spiniger* of dogs and one from ked of sheep (Table 5 and Fig. 3). All these 3 new *Acinetobacter* spp. demonstrated low nucleotide homology with reference *rpoB* sequence in the GenBank and low bootstrap value in the *rpoB* phylogenetic tree. Results of our study suggest the presence of specific *Acinetobacter* species in lice and keds of domestic animals unlike *A. baumannii* in human lice. We believe that additional in depth epidemiological studies involving other species of lice and ectoparasites of different animal species from vast areas in the world are needed as has been done during the last decade for *Bartonella* species, ectoparasites and their animal hosts [52]. Further studies are also required to isolate and determine the human health significance of the new *Acinetobacter* spp. detected in the lice and keds from animals.

To our knowledge, this study is the first to report the presence of DNAs from different *Acinetobacter* spp. in various species of lice collected from domestic animals and in flies collected from sheep. Our study demonstrates that *Acinetobacter* spp. are not only common as hospital pathogens and in human lice but they can also be detected in the ectoparasites of animals. Our findings suggest that synanthropic animals and their ectoparasites might play a role to increase the risk of human exposure to zoonotic pathogens and could be a source for *Acinetobacter* spp. infections in humans. However, additional epidemiological data are required to justify the significance of this finding and to determine whether ectoparasites from animals can act as environmental reservoirs and play a role in spreading these bacteria to both animal and human hosts.
